# Editorial: Animal Models of Anxiety and Depression: Exploring the Underlying Mechanisms of Sex Differences

**DOI:** 10.3389/fnbeh.2022.961825

**Published:** 2022-07-18

**Authors:** Laura B. Tucker, Mario G. Oyola, Deborah Suchecki, Nikolaos Kokras

**Affiliations:** ^1^Preclinical Behavior and Modeling Core, Uniformed Services University of the Health Sciences, Bethesda, MD, United States; ^2^Naval Medical Center Camp Lejeune, Jacksonville, FL, United States; ^3^Department of Psychobiology, Universidade Federal de São Paulo, São Paulo, Brazil; ^4^First Department of Psychiatry, Eginition Hospital, Medical School, National and Kapodistrian University of Athens, Athens, Greece; ^5^Department of Pharmacology, Medical School, National and Kapodistrian University of Athens, Athens, Greece

**Keywords:** sex differences, females, anxiety, depression, gender differences

Anxiety and depression carry a significant burden and disproportionally affect more women than men (Balta et al., 2019). Moreover, men and women differ in symptomatology and responses to psychotropic agents, highlighting the need for a better understanding of the mechanisms leading to these sex differences (Butlen-Ducuing et al., 2021). As the exact neurobiology of these disorders still eludes us, animal models are routinely employed to study anxiety and depression. Although male animals have been traditionally used in pre-clinical studies, the inclusion of both sexes, as recently dictated by NIH policies (Clayton and Collins, 2014), presents an opportunity to explore sex differences in the biological underpinnings, contributions to stress, and other influences that may underlie emotional dysregulation and abnormal performance at behavioral endpoints. Emerging evidence indeed uncovers significant sex differences in most animal models of depression and anxiety, either at baseline or following treatment. Such differences may have substantial implications for translating preclinical to clinical research (Kokras and Dalla, 2014, 2017). Unfortunately, animal models have also yielded inconsistent results and often report greater anxiety- or depressive-like symptoms in male than in female animals or do not show sex differences. For example, a recent study found that the frequently-employed chronic unpredictable mild stress model was more likely to induce depressive-like behaviors in male than in female rats (Iqbal et al., 2020).

In this Research Topic, experiments performed by Eltokhi et al. at two different developmental stages during adolescence revealed strain but no sex differences in a set of depression-related tests, including tail suspension, sucrose preference, and forced swim tests. However, when tested in the anxiety-related hyponeophagia test, male and female mice behaved differently. In continuation, Pitzer et al. showed that, like in adolescent, neither adult C57BL/6N, DBA/2 or FVB/N present significant baseline sex differences in behavioral tests measuring immobility in tail suspension and forced swim tests, as well as anhedonia in the sucrose preference test. However, adult male and female mice showed significantly different results in the baseline apathy-like behaviors depending on the investigated strain. These studies by Eltokhi et al. and Pitzer et al. provide a good baseline characterization of the C57BL/6N, DBA/2 and FVB/N mouse strains regarding the absence of sex differences at two different ages in certain tests. Moreover, they highlight the importance of considering several factors, such as strain, age, type of tests, and behavioral outcome when studying sex differences. Such an approach that avoids overlooking critical factors that can influence the planning, conduct and results of studies can increase the reproducibility of preclinical research (Sil et al., 2021). As Eltokhi et al. and Pitzer et al. note, inconsistencies of results between different laboratories investigating rodent models of depression and anxiety call for better standardization and normalization when designing experiments exploring sex differences. In this context, it is important to use appropriate animal models that reproduce specific aspects of the complex clinical manifestations at the behavioral and molecular levels. In this Research Topic Touchant and Lebonte summarize findings from animal models and discuss genome wide transcriptional strategies for such complex clinical manifestations. Such strategies may provide crucial insights into the neurobiological underpinnings of these diseases and the basis of sex-specific molecular responses in experimental animals and humans. Another equally important issue is screening for new psychotropic drugs using both sexes. In this context, Yin et al. studied the effects of Yueju-Ganmaidazao Decoction (YG), a substance with potential antidepressant actions, in relation to NO-cGMP signaling. They found that both YG and escitalopram induce antidepressant-like behavioral responses in both sexes. However, both drugs enhanced CaMKII-nNOS expression in the hippocampus of female mice, in opposition to what was observed in male mice, despite the same behavioral antidepressant response in both sexes. This concept highlights another issue in studying sex differences: that the same behavioral response can be observed in both sexes, but the underlying neurobiological processes that lead to the same behavioral response in male and female brains are not necessarily identical, as also noted previously (Kokras et al., 2011).

On the other hand, brain regions such as the hippocampus and the prefrontal cortex have long been implicated in the neurobiology of stress, anxiety and depression (Duman et al., 1997). Emerging preclinical data identify prominent sexual divergence in these regions as reviewed in this Research Topic by Wallace and Myers, who suggest that chronic stress has sex-specific effects on the rodent infralimbic cortex excitatory/inhibitory balance that may account for sex differences in the prevalence and course of mood disorders. Moreover, McNamara et al. studied sex differences in limbic responses after shock wave exposure, which resulted in a transient blood-brain barrier (BBB) breach of variable severity. Subsequent testing showed sex differences in various behavioral tests of anxiety and depression and in c-Fos expression post-injury. The authors suggest that the increased vulnerability of women to post-traumatic stress disorder could be related to the mild effects of post-injury behavioral and neuronal effects that they observed in the female mice in their study. Sex differences in the BBB is an emerging subject of interest. Dalla et al. summarized preclinical and clinical findings on how sex and sex hormones can influence the activity of BBB transporter systems. They concluded that accumulated evidence supports the existence of several sex differences in expression and activity of BBB transport proteins, which are also modulated by gonadal hormones. As is the case with the BBB, to understand sex differences following stress, we must consider how all cell types within the central nervous system are involved. Indeed Wegener and Neigh in their review discuss the effects of stress and sex steroids on astrocytes and oligodendrocytes. They conclude that studies exploring the mechanisms by which glia are altered by stress and steroids will provide insight into sex differences in animal models. In a similar context Michailidis et al. used the spared nerve injury (SNI) model of neuropathic pain and noted that behavioral depressive-like responses were first observed at different time points in male and female animals. They then proceeded to immunohistochemical analysis and showed that microglial cells were more numerous in female mice in the contralateral ventral anterior cingulate cortex, suggesting that different patterns of glial cell activation may be associated with pain processing and affect in male and female animals. As Gaspar et al. note in their study, microglia, the immune cells of the brain, are involved in the stress-related neuronal and behavioral response, and thus contribute to the development of stress-related psychopathologies. The authors found that following short-term unpredictable chronic mild stress, both male and female rats showed anxiety-like behavior. However, after longer term chronic stress, male animals demonstrated depression- and anxiety-like behaviors but females demonstrated only the later. Subsequent investigation showed that microglia cells in the dorsal hippocampus and in the nucleus accumbens were found to adapt differently according to duration of stress, brain region studied, and, importantly, sex of the animals.

Finally, early life adversity in humans and rodents is associated with sex-specific emergence of anxious and depressive behaviors, and Ellis and Honeycutt summarized in this Research Topic such findings and suggest the possibility of a combined role of sex hormones and calcium-binding protein parvalbumin expressing neurons driving differences in behavioral outcomes associated with affective dysfunction following early life adversity. The overall message from this Research Topic (Figure 1) is that sex differences are observed in many different levels of preclinical research, and as the field of sex differences in neuroscience emerges and accumulates more data, a better understanding of such differences may improve our understanding of depression and anxiety, and lead to better treatments for both diseases.

**Figure 1:**
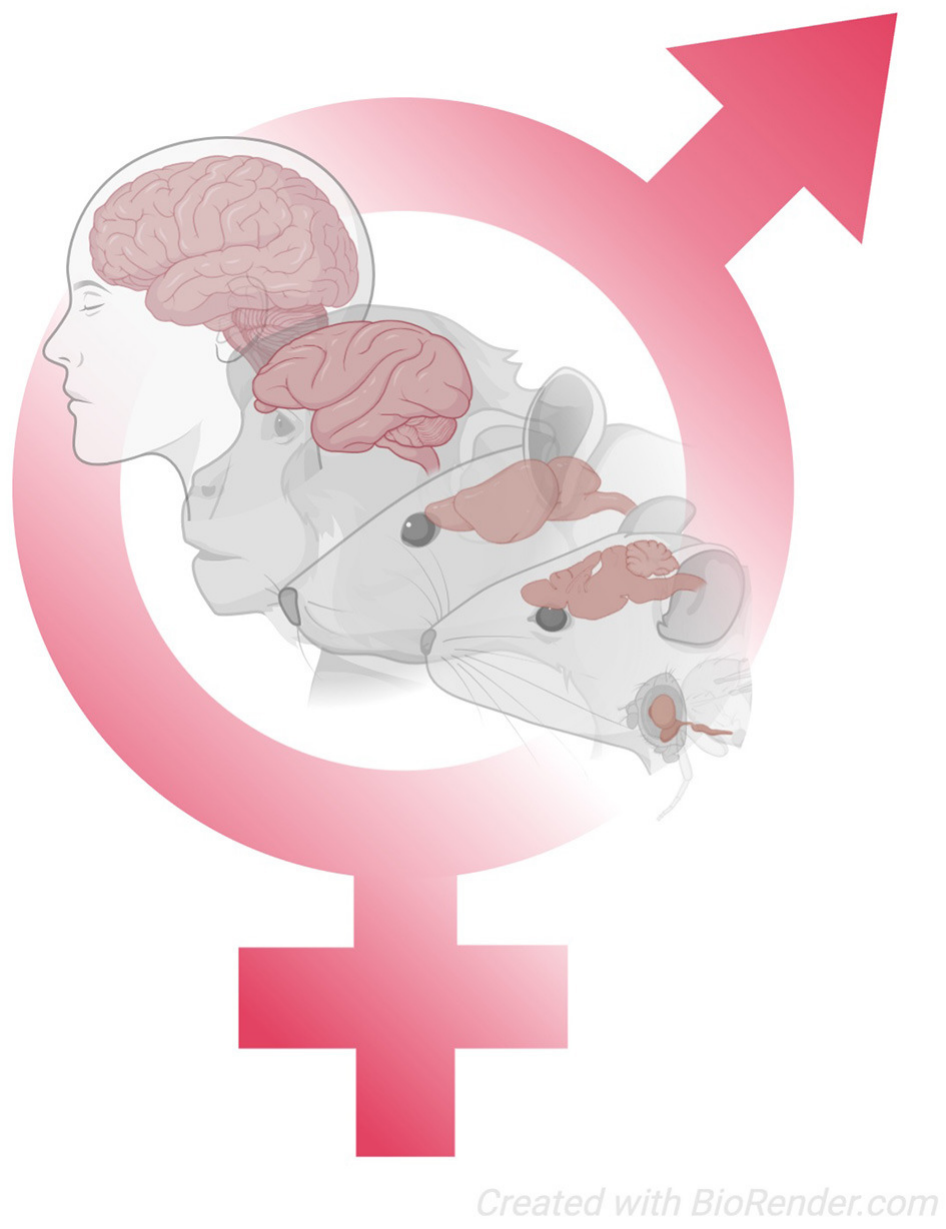
Animal models of anxiety and depression: Exploring the underlying mechanisms of sex differences. Image created with BioRender.com.

## Author Contributions

NK compiled the first draft. All authors made a substantial, direct, and intellectual contribution to the work and approved it for publication.

## Funding

NK research is co-financed by Greece and the European Union (European Social Fund—ESF) through the Operational Programme Human Resources Development, Education and Lifelong Learning in the context of the project-Reinforcement of Postdoctoral Researchers—2nd Cycle (MIS-5033021), implemented by the State Scholarships Foundation (IKY).

## Author Disclaimer

The opinions, interpretations, conclusions and recommendations are those of the authors and are not necessarily endorsed by the U.S. Army, Department of Defense, the U.S. Government or the Uniformed Services University of the Health Sciences. The use of trade names does not constitute an official endorsement or approval of the use of reagents or commercial hardware or software. This document may not be cited for purposes of advertisement.

## Conflict of Interest

The authors declare that the research was conducted in the absence of any commercial or financial relationships that could be construed as a potential conflict of interest.

## Publisher's Note

All claims expressed in this article are solely those of the authors and do not necessarily represent those of their affiliated organizations, or those of the publisher, the editors and the reviewers. Any product that may be evaluated in this article, or claim that may be made by its manufacturer, is not guaranteed or endorsed by the publisher.
